# Earth as a Topical Application in Surgery

**Published:** 1887-08

**Authors:** 


EARTH AS A TOPICAL APPLICATION IN SURGERY, being a Full Exposition of its Use in all the Cases Requiring Topical Applications admitted in the Men's and Women's Surgical Wards of the Pennsylvania Hospital during a period of six months in 1869. By Addinell Hewson, M. D. Second edition, with four Photo-relief Illustrations. Philadelphia: The Medical Register Co.

The length of time required to exhaust the first edition of this work shows how slowly the idea of using earth in surgery has affected the medical mind. Popularity is no measure of success, neither does it necessarily involve inferiority. But it does not follow, as many seem to think, that unpopularity is an indication of excellence. The only criteriom is, "By their fruits ye shall know them." There is no doubt that Dr. Hewson has satisfactorily shown that earth is often useful in surgery; that it is an efficient deodorizer; that it acts beneficially in relieving pain and promoting healing action. But its results do not demand that it should ever supplant the antiseptics. As well expect to replace railroads by stage-coaches. However, this book is interesting, for intelligent experiment is ever attractive to the lover of truth, and it is useful, in that it adds to the variety of the physician's remedies in emergencies. It reminds one, though, of old Mrs. Mulligan, who bought a door-plate bearing the name of Thompson, because she might have a daughter, and that daughter might marry a man named Thompson. There is too much of indisputable and indispensable knowledge to learn in this day and time to occupy one's time on such a subject. H. H.

